# Mandible shape variation and feeding biomechanics in minks

**DOI:** 10.1038/s41598-022-08754-4

**Published:** 2022-03-23

**Authors:** Eloy Gálvez-López, Philip G. Cox

**Affiliations:** 1 PalaeoHub, Department of Archaeology, University of York, Wentworth Way, Heslington, York, YO10 5DD UK; 2 Hull York Medical School, University of York, Heslington, York, YO10 5DD UK

**Keywords:** Biomechanics, Conservation biology

## Abstract

European and American minks are very similar in ecology, behavior and morphology. Both species hunt terrestrial vertebrates and aquatic prey, but the American mink is a more generalist predator which, among other factors, allows it to outcompete the European mink in areas where it has been introduced. We used 3D geometric morphometrics and estimates of muscle mechanical advantage to assess the degree of variation in mandibular morphology, and to determine whether such variation reflects dietary differences between the two species. The three main axes of variation represented interspecific differences, a common allometric trajectory between species and sexes, and the interspecific effect of sexual size dimorphism, with males having overall stronger bites than females. Differences in mandible shape and biomechanical parameters suggest that American minks are better equipped for preying on terrestrial vertebrates, while the features seen in European mink could be related to tougher prey, fish capture, or both. Additionally, within each species, the larger specimens of each sex present indicators of a higher percentage of terrestrial prey in their diet. These results indicate a low potential dietary overlap between both species, suggesting that factors other than prey competition may have a role in the decline of the European mink.

## Introduction

Understanding what an animal eats in the wild not only sheds light about its role in the ecosystem and its interaction with other species, but also provides important information towards conservation strategies, management plans and maintenance of animals in captivity^1,2^. However, due to the varying outcomes and limitations of the techniques employed in diet studies (field observation, scat analyses, stomach contents, DNA metabarcoding; e.g.^3–5^) and potential seasonal, regional or individual variation, a large number of these studies are required to accurately assess an animal’s diet^6–15^. Alternatively, ecomorphology studies use an animal’s anatomy to infer its ecology (e.g. diet^16^), which allows us to understand both what prey items an animal is capable of consuming and why, instead of observing what is it eating at a precise point in time.

Mandible morphology has been used extensively to explore dietary adaptations in carnivorans^17–21^, since feeding is the main function of this skeletal component^22^. Additionally, mandible morphology has been shown to be evolutionarily more plastic, reflecting dietary adaptations more accurately than the cranium, whose morphological evolution must respond to conflicting functional demands^23–25^. However, in the last decades, the consolidation of geometric morphometric methods has resulted in an increase in this type of study, which have revealed that mandible shape (i.e., all its geometric features except for size, position, and orientation) in carnivorans is not only related to function (i.e., mastication), but to a complex interaction of factors such as evolutionary history, body size, sexual dimorphism, diet and, in carnivorous species, prey size^16,23,26–31^.

Regarding dietary adaptations, several traits in mandible shape have been identified as characteristic of different diets in Carnivora. For instance, carnivorous carnivorans have a relatively shorter corpus and coronoid process, a reduced crushing molar region and an enlarged slicing carnassial region^17,26–29^. In contrast, a relatively longer corpus seems to be indicative of piscivory^21^, and durophagous carnivorans present markedly tall coronoid processes, increased distance between condylar and angular processes, and a thickened corpus, particularly posteriorly to the crushing teeth^18,21,23^. Additionally, enlarged canines and a deep anterior corpus are associated with powerful killing bites^18,27,28^. With that in mind, we wished to determine whether morphological differences would be identifiable between closely related species and, if so, whether it would reflect relatively small dietary differences between those species.

The study species for this analysis were the European mink (*Mustela lutreola* Linnaeus, 1761) and the American mink (*Neovison vison* Schreber, 1777), two small mustelids with very similar phenotypes and ecologies. Both minks are semiaquatic species adapted to riverine habitats, where they hunt both terrestrial vertebrates and aquatic prey^32^. However, the extent of dietary overlap or prey competition between both mink species is unclear due to the wide regional and seasonal variation of main prey in American mink^10,15,33^. This convergent ecomorphology led to extensive competition between both species when the American mink spread throughout Europe after escaping from fur farms and even being deliberately released to the wild to establish populations from which to hunt^34^. However, the larger size, more generalist diet and greater tolerance for anthropized environments^32,35^, among other factors, allowed the invasive species to vastly outcompete the native mink, which has been classified as Critically Endangered on the IUCN Red List since 2011^36,37^. Furthermore, the results of a recent study on cranial shape in minks hinted at dietary differences between both species^38^, and also suggested that female European mink could be being displaced to an increasingly narrower, poorer diet when both species coexist. Thus, studying mandible shape variation in both species will provide further information on their dietary capabilities and feeding biomechanics, and will help clarify whether female European mink are indeed the losers in inter- and intra-specific prey competition. We are aware that two-species studies are not adequate to define adaptations to environmental factors such as diet (see^39^). Thus, dietary adaptations identified in multispecies studies on Carnivora^16–24,26–29^ are used here as indicators of dietary capabilities.

This study aims then to (1) describe and compare mandible shape variation in European and American minks; (2) analyze how factors such as size, sexual dimorphism, and their interactions, affect shape variation in both mink species; and (3) assess whether mandible shape differences between European and American minks could be related to potential differences in trophic specialization (i.e., dietary indicators). As was observed with cranial morphology, there has been no previous comparison of mandible morphology between these two mink species, so we can only hypothesize potential differences. Based on cranial shape results^38^ we expect that: (1) Both species will be clearly distinguishable based on mandible shape alone; (2) Both size and sex will have a significant effect on mandible shape, with significant factor interactions expected between species and size (i.e., different intraspecific allometries) and species and sex (i.e., sexual dimorphism in mandible shape); and (3) Some trophic specialization will be observed both between species (e.g. indicators of piscivory in European mink and durophagy in American mink) and between sexes (e.g. indicators of stronger killing bites in males). Previous studies on mandible shape variation in both carnivorans as a whole^26^ and other mustelid species^28,30,40^ support a significant effect of size and sex on mandible shape and different intraspecific allometric trajectories for both mink species. However, evidence for sexual dimorphism in mandible shape is scarce, having only been described so far for *Enhydra lutris*^30^ and *Mustela sibirica*^28^.

## Results

### Shape variation

The results of the Procrustes ANOVAs revealed that mandible shape is significantly different between European and American minks (F = 39.170; *p* < 0.001), and that size explains 6.29% of total shape variation in both species (F = 11.486; *p* < 0.001). Additionally, sex also had a significant effect on mandible shape (F = 8.526; *p* < 0.001). Thus, since all factors had a significant effect on mandible shape, all the potential factor interactions were analyzed (Table 1). Both sexual dimorphism in shape (spp*sex interaction) and interspecific sexual allometry (sex*size interaction) had a significant effect on mandible shape, which was further explored using phenotypic trajectory analysis (PTA) (Table 2). Significant differences in mandible shape were found for all pairwise comparisons by species and sex (Table 1), and PTA revealed that shape differences between males and females of each species are different in orientation but not magnitude (Table 2). Similarly, PTA of interspecific sexual allometry indicated that both sexes follow allometric trajectories with similar magnitudes but different orientations (Table 2).

**Table 1:** Factor interactions in mandible size and shape.

Intraspecific allometry		Interspecific sexual allometry	
spp	*1.186 (0.238)*	sex	3.170 (0.001)
CS	2.942 (< 0.001)	CS	3.159 (0.001)
spp*CS	*1.230 (0.207)*	sex*CS	2.800 (0.004)

Sexual dimorphism			
	Shape	CS	PC2
spp	17.940 (< 0.001)	*0.179 (0.680)*	*0.021 (0.884)*
sex	2.571 (< 0.001)	20.277 (< 0.001)	*2.370 (0.131)*
spp*sex	2.076 (0.010)	14.671 (< 0.001)	7.356 (0.007)
Pairwise			
Mlu.F–Mlu.M	0.030	0.005	*0.356*
Mlu.F–Nvi.F	0.001	*0.776*	*0.892*
Mlu.F–Nvi.M	0.001	0.001	0.001
Mlu.M–Nvi.F	0.001	0.001	*0.181*
Mlu.M–Nvi.M	0.001	0.001	0.001
Nvi.F–Nvi.M	0.001	0.001	0.001

Results from the Procrustes ANCOVAs for species and sex with centroid size as covariate, and of the two-way Procrustes ANOVA (mandible shape) and non-parametric ANOVA (centroid size) with species and sex as categorical variables. For each factor, Goodall’s F values are provided together with its associated *p* value (in brackets), with non-significant *p* value in italics. Results of post-hoc pairwise tests used to assess differences between species-sex pairs are also provided.

CS, centroid size; F, female; M, male; Mlu, European mink; Nvi, American mink; spp, species; sex*CS, interaction between sex and size; spp*CS, interaction between species and size; spp*sex, interaction between species and sex.

**Table 2:** Phenotypic trajectory analyses.

	Magnitude		Z	Angle	Z
sex*CS	F = 0.0022	M = 0.0026	*0.132 (0.373)*	65.67º	3.723 (0.003)
spp*sex	Mlu = 0.018	Nvi = 0.024	*1.063 (0.150)*	54.15º	3.231 (0.004)

Results from the trajectory comparison in sexual dimorphism (spp*sex) and sexual allometry (sex*CS) of mandible shape in minks. For each comparison, values of the Z statistic are provided together with its associated *p* value (in brackets), with non-significant *p* value in italics. Abbreviations as in Table 3.

**Figure 1:**
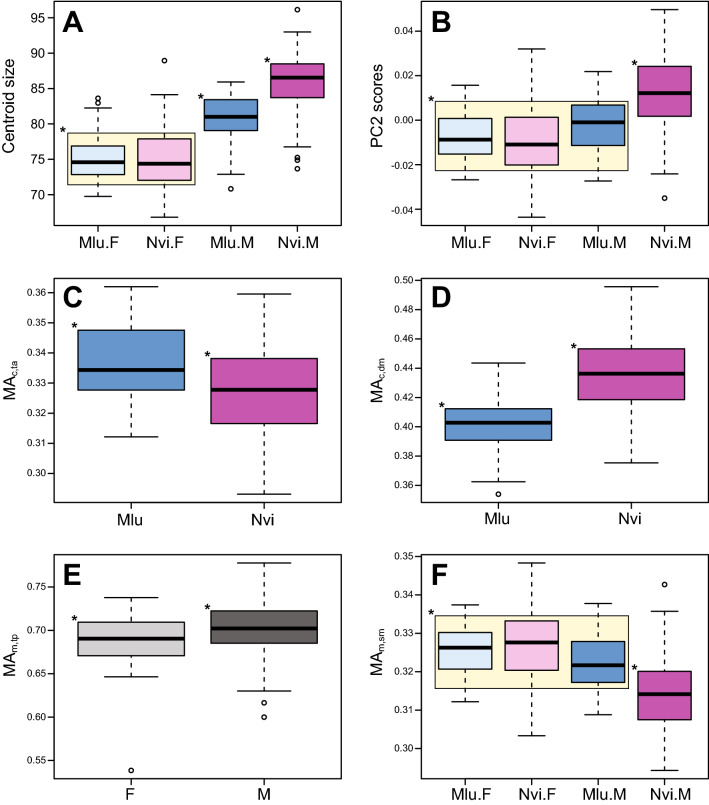
Sexual dimorphism in mandible size and jaw biomechanics. Boxplots for centroid size (**A**) and PC2 scores (**B**) by species and sex, and for the MAs of the anterior temporalis (**C**) and the deep masseter (**D**) by species, of the posterior temporalis by sex (**E**) and of the superficial masseter by species and sex (**F**). Bold line denotes the median (quartile 2; Q2), while the box represents interquartile range (IQR: Q1 to Q3) with whiskers extending 1.5 times IQR. An asterisk on the top left corner of a box indicates that the mean for that group is significantly different from all other asterisks in the panel. In all plots by species and sex, all groups not different from each other are placed within a yellow box. Abbreviations: F, female; M, male; Mlu, European mink; Nvi, American mink.

Despite the overall larger body size of American mink, no significant differences in centroid size (CS) were found between species (F = 2.869; *p* = 0.091), although males were significantly larger than females in both species (F = 14.671; *p* < 0.001). Note, however, that the percentage of mandible shape variation explained by size was different for both species (Mlu: 5.98%; Nvi: 9.26%; *p* < 0.001 in both cases). Pairwise CS comparison by species and sex were significant for all pairs except females of both species (Table 1; Fig. 1A).

**Figure 2:**
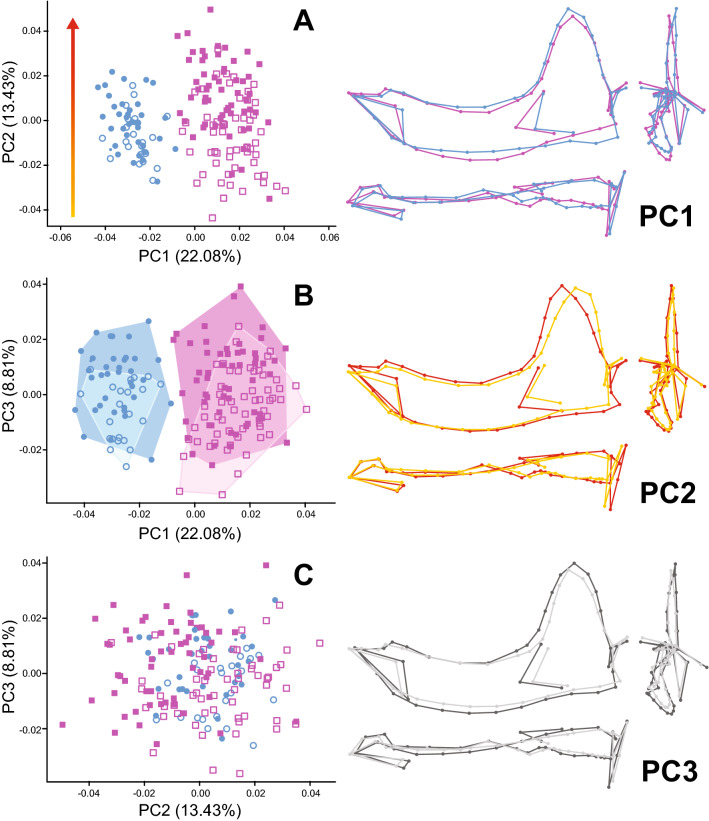
Principal component analyses of mandible shape variation in minks. (**A**) PC1 vs PC2, (**B**) PC1 vs PC3, (**C**) PC2 vs PC3. The percentage of total variance explained by each principal component is given in parentheses. Key: blue circles, European mink (Mlu); pink squares, American mink (Nvi); solid symbols, males; open symbols, females. Minimum convex polygons for males (darker shades) and females (lighter shades) of each species are drawn on panel (**B**). The wireframes on the right illustrate the shape variation along each PC from the lowest (PC1: blue/Mlu; PC2: yellow/small; PC3: light grey/female) to the highest score (PC1: pink/Nvi; PC2: red/large; PC3: dark grey/male).

The first three principal components (PCs) explained 44.32% of total shape variation (Fig. 2). PC1 separated both species without any overlap: higher values represented American mink and lower values European mink (Fig. 2A,B). In agreement with this, the non-parametric ANOVA of PC1 by species indicated that interspecific shape differences account for 81.45% of PC1 variation (F = 750.70; *p* < 0.001). According to the shape changes along PC1, European mink presented the following morphology relative to American mink (Fig. 2, Fig. S1): a taller and more anteriorly expanded coronoid process; a straighter and slightly shorter corpus; a ventrally displaced angular process and fossa; a caudally retracted masseteric fossa; and a shorter toothrow (with slightly longer canines and smaller p2, p4 and m2). Remarkably, both the size of m1 and the relative proportion of its shearing and crushing aspects remained constant along PC1. While sex only explained 3.4% of shape variation along PC1 (F = 5.946; *p* = 0.019) and size had no significant effect on it (F = 0.127; *p* = 0.721), a significant interaction effect between those factors was recovered for PC1 (F = 10.200; *p* = 0.002). However, we suspect that this is an artifact caused by the slightly larger PC1 values of male American minks (Fig. S2A), as all intraspecific sexual allometries were parallel (i.e., no significant triple interaction for PC1, spp*sex*CS: F = 0.920; *p* = 0.345; Fig. S2B).

PC2 was mainly related to size, which explained a third of the shape variation along this axis (33.07%; F = 84.493; *p* < 0.001). Unsurprisingly, since males had significantly larger mandibles than females, sex also was significantly related to PC2 (17.76%; F = 36.288; *p* < 0.001). Interspecific differences in PC2 values were marginally significant (2.4%; F = 4.236; *p* = 0.043), but the interaction between species and sex was strong, somewhat mirroring the results for sexual dimorphism in mandible centroid size (Table 1; Fig. 1B). Finally, all factor interactions with size as covariate (i.e., allometries) were not significant, suggesting that PC2 represents a common trend in intraspecific allometry (spp*CS: F = 1.753; *p* = 0.186), interspecific sexual allometry (sex*CS: F = 0.702; *p* = 0.403; Fig. S2C), and intraspecific sexual allometry (spp*sex*CS: F = 0.071; *p* = 0.792; Fig. S2D). Thus, as mandible size (and hence PC2 values) increases, the following shape changes can be observed (Fig. 2, Fig. S3): the coronoid process shifts anteriorly and widens at its base; the angular process expands ventrocaudally, with its tip shifting medially; the corpus expands dorsally, particularly in its anterior part; the masseteric fossa expands anteroventrally; and the toothrow becomes shorter, as all teeth after p2 become smaller while the canine and p2 are slightly larger.

Finally, PC3 was significantly related to both sex and size, with both factors explaining about 12% of this axis shape variation (F = 22.712 and F = 25.077 respectively; *p* < 0.001 in both cases). Since males of both species had similar and larger PC3 values than females of both species (which also presented similar values), and the interaction between sex and size was not significant (F = 1.058; *p* = 0.309), PC3 seems to represent shape changes associated with sexual dimorphism in mandible size which are common to both species. Relative to small females, the larger males present (Fig. 2, Fig. S4): a wider and slightly caudally oriented coronoid process; a more robust corpus (ventral midline expands ventrally, border of masseteric fossa shifts laterally); an anteriorly expanded masseteric fossa; and a shorter toothrow which accommodates larger canines, smaller premolars and similarly sized molars (including shearing/crushing proportion) by becoming more concave.

### Muscle biomechanics

MA values for each specimen can be found in Table S1, while the results for all the ANOVAs can be found in Table S2. When estimated from the original specimens, MAs at the anterior teeth significantly increased with size, while at the carnassials the MA of the posterior temporalis was not related to size and that of the superficial masseter significantly decreased with size. Between species, all MAs were higher in European mink (Fig. 1C) except for the MA of the deep masseter, which was higher in American mink (Fig. 1D). Between sexes (Fig. 1E), males had significantly larger MAs for all muscles but the superficial masseter, although sexual dimorphism (spp*sex) was not significant for any MA. Significant differences in intraspecific allometry (spp*CS) were found for the MA of the anterior temporalis, which scaled faster in European mink than in American mink (Fig. S2E). In terms of interspecific sexual allometries (sex*CS), the MA of the anterior temporalis scaled faster in females (Fig. S2F). No significant differences in intraspecific sexual allometries were found for any of the variables.

In agreement with PC1 representing interspecific differences, the ANOVAs for the MAs calculated on PC1 configurations revealed significant differences between species, which in turn mirrored the interspecific differences obtained for the original specimens (Fig. 1C,D): all MAs were higher in European mink except for the MA of the deep masseter. As with shape variation along PC1, sex and interspecific sexual allometry also had a significant effect, which again might be an artifact caused by the higher PC1 scores of male American mink (Fig. S2A, B). Similarly, results for the MAs calculated on PC2 configurations mirror those of CS and PC2 scores, with significant sexual dimorphism (spp*sex) driven by the larger size of male American mink (Fig. 1A,B). The sole exception was the MA of the superficial masseter, which reversed this trend (Fig. 1F). Finally, MAs calculated on PC3 configurations significantly increased with CS and were thus higher in males than in females.

## Discussion

This is the first study analyzing mandible shape in both mink species and, together with a previous study on their cranial shape^38^, it has revealed how small morphological differences in highly similar species can lead to substantial biomechanical differences (see breakdown below). As with cranial shape, mandible shape in minks is influenced by the complex interaction of size and sexual dimorphism both at the inter- and intraspecific levels. However, while in cranial shape both species had divergent shape allometries and parallel interspecific sexual allometries, the opposite was true for mandible shape.

Differences in mandible shape between European and American mink were summarized by PC1 (Fig. 2, Fig. S1) and can be mainly related to muscle size and jaw biomechanics (i.e., in-levers and out-levers). The relatively taller and slightly wider coronoid process of European minks suggests a relatively larger temporalis muscle, while the anteriorly expanded masseteric fossa of American mink is indicative of a relatively larger masseter complex^17,22,25^. The relatively enlarged angular process of European mink provides a larger attachment area for the superficial masseter, with both mink species having a distinctive fossa on the lateral side of the angular process where this muscle attaches. This angular fossa is not present in European polecats (Gálvez-López, pers. obs.), part of the sister clade to European mink^41^.

Regarding jaw biomechanics, the particular morphology of the American mink illustrates the compromise between maximizing both bite force efficiency and increased gape. The MAs for all masticatory muscles were higher in European mink due to their relatively longer in-levers (and also shorter out-levers if measured on PC1 configurations), with the exception of the MA of the deep masseter which was considerably higher in American mink (Table S2; Fig. 1D). These findings indicate that American mink exhibit features that allow them to produce larger forces at wide gape, which is particularly useful for holding and killing terrestrial vertebrates^22,42^. In agreement with this, a short moment arm of the superficial masseter (as observed in American mink) has been associated with increased gape in other mammals^43^. It is also worth noting that low MAs for the posterior temporalis and superficial masseter have also been associated with fish capture, as they indicate a relatively longer mandible relative to the muscle in-levers, which in turn allows the mouth to close faster when trying to catch elusive prey underwater^21^. In contrast, the characteristic features of European mink are indicative of stronger bites at the carnassials, which would allow them to cut through relatively tougher tissues and also to crush harder objects (e.g. shells of aquatic prey). Favoring carnassial over anterior bites could also be advantageous to feeding on fish. Mink catch fish underwater by grabbing them by the fins or back with their anterior teeth, and then dragging them to the surface where they are processed using cheek (carnassial) bites (Gálvez-López, pers. obs.).

In our previous study on cranial shape in mink^38^, morphological differences between both species indicated relatively larger muscle volumes overall in the American mink (temporalis: more developed sagittal and nuchal crests, narrower braincase; masseter: longer and more curved zygomatic arches, larger infratemporal fossa), which suggested that bite forces both at the anterior dentition and at the carnassials were larger in this species. However, when combined with the MA results from this study on mandible shape, the relationship between muscle volume and force production becomes less straightforward. In the case of the European mink, the relatively smaller temporalis has a larger attachment site on the mandible (i.e., a broader and taller coronoid) and becomes more efficient (i.e., has higher MAs) due to the relatively longer in-lever. Similarly, in the American mink the effective length of the superficial masseter is increased by the marked curvature of the zygomatic arches, which mitigates the dorsal displacement of the angular process. However, the efficiency of the relatively larger temporalis is diminished by a smaller coronoid (i.e., reduced attachment area and shorter in-levers). The remaining differences in cranial morphology align with differences in mandible shape. Namely, the relatively broader zygomatic arches of the European mink support a strong superficial masseter, while the larger infratemporal fossae of American mink account for their enlarged deep masseter. On a final note, another finding common to both cranial and mandible shape was the relatively larger crushing dentition of American mink.

Thus, after combining the results of cranial and mandible shape, it appears that, while the characteristic features of European mink indeed allow stronger carnassial bites, American mink present morphological indicators of both strong killing bites at wide gapes and powerful carnassial bites with a marked crushing component.

The allometric effect on mandible size common to both species was represented by PC2 (Fig. 2, Fig. S3), which complements the common allometric trend recovered for both mink species in cranial shape^38^. The relative expansion of the masseteric fossa and the angular process with increasing size suggests that larger mink present a larger masseter complex. However, most of the allometric shape changes are related to muscle in-levers and out-levers. With increasing size, the length of both the out-lever at the anterior teeth and the in-levers of its related muscles (anterior temporalis, deep masseter) increases (Table S2), but the in-levers scale faster than the out-lever (Table S2). Thus, the mechanical advantages of both muscles at the anterior teeth also increase with size (Table S2), indicating that larger mink have markedly stronger and more efficient killing bites (particularly true for the deep masseter, which also becomes larger with size). This, together with their relatively larger anterior dentition (both in the mandible and the cranium) and taller anterior corpus, can be related to feeding on larger prey as size increases (i.e., stronger bites to perforate tougher skulls and hold onto stronger struggling prey, which would also require more robust teeth and corpora to resist the stresses placed on them). Similar features have been described for felids^18^, which also kill prey in this way^22,32^.

Note, however, that one of the shape changes along PC2 does not accurately reflect the common allometric pattern: the lever arm of the superficial masseter, which slightly decreases along PC2 (Fig. 2; Table S2) and results in a decrease of the mechanical advantage of the superficial masseter and hence bite force at the carnassials along this axis (Table S2). In contrast, this lever arm significantly increases with size in the original specimens (Table S2), in agreement with the common allometric trend in cranial shape suggesting stronger bites at all teeth with increasing size^38^. A likely explanation for this phenomenon is that the common allometric trend is being confounded with interspecific shape differences, as American mink have significantly shorter superficial masseter in-levers than European mink (Fig. 1F; Table S2) yet their males are significantly larger than all other specimens (Fig. 1A). As mentioned above, the relative decrease in MA might reflect the trade-off between producing strong bite forces at the anterior teeth and having a wider gape to capture larger prey^43^, both of which are heavily supported by other morphological features in this common allometric trend.

Sexual dimorphism in mandible shape was significant both within each species, and when grouping sexes from both species together. In her study of Palearctic mustelids, Romaniuk^28^ also found evidence for interspecific sexual dimorphism in mandible shape, but within species it was only significant for the Siberian weasel (*Mustela sibirica*). The different results for the European mink in that study might be related to its smaller sample. Note, however, that Hernández-Romero et al.^40^ did not find evidence for sexual dimorphism in mandible shape within Neotropical otters (*Lontra longicaudis*) even though their sample sizes were equivalent to those in the present study.

Overall, the results of the present study reveal that mandible shape differences between males and females are the consequence of a complex interaction between sex and size at both inter- and intraspecific levels. For instance, each sex in each species has a mandible shape significantly different from each other (Table 1), but allometric shape changes within each of them are similar (except maybe female American mink; Fig. S5A). Additionally, while trajectory analysis indicates that the degree of sexual dimorphism in mandible shape is similar within each species, the specific differences between sexes are different in each species (i.e., same magnitude, different orientation; Table 2, Fig. S5B). While at the interspecific level, male and female mandible shapes change differently with increasing size even though the change per unit size is similar in both sexes (Tables 1, 2; Fig. S5C,D), and some of the allometric changes are common to both species and sexes (see section above; PC2 in Fig. 2). Finally, another set of shape changes related to sexual dimorphism and common to both species are those related to sexual dimorphism in mandible size, illustrated by PC3 (Figs. 2, Fig. S4).

Shape changes related to sexual dimorphism in size are represented along PC3 and can be related to an overall increase in bite force (i.e., at all teeth), as higher scores on this axis correspond to increased muscle attachment areas and longer in-levers (taller and wider coronoid, anteriorly expanded masseteric fossa, ventrally expanded angular process), shorter out-levers (particularly at the anterior teeth), and a more robust corpus (dorsoventrally and mediolaterally expanded). This interpretation of shape changes along PC3 is supported by the results of the ANOVAs on the lever arms and MAs measured on the PC3 configurations (Table S2). These variables were only related to sex and size, with female mink having longer out-levers and male mink presenting longer in-levers and higher MAs, while out-levers decreased with increasing size and in-levers and MAs increased in both sexes (no significant interaction between sex and size indicates parallel allometric trajectories in both sexes). This trend is consistent with the common sexual allometry described for cranial shape, which suggested that larger males have bigger masticatory muscles than smaller females and thus produce higher bite forces^38^. Additionally, even though the relative length of the toothrow decreases, the size of the canine markedly increases and there is no change in molar size or the relative proportions in its shearing and crushing regions. Although this might be interpreted as reinforcing the canines to cope with killing larger prey while maintaining an otherwise similar dietary regime^20^, it is worth noting that larger canines have been long described as a feature of sexual size dimorphism in mustelids^19,44,45^.

In terms of interspecific differences in sexual allometry, with increasing size the following shape changes were observed in females but not in males (Fig. S5C): a dorsoventrally more robust corpus, a ventral expansion of the angular process, longer in-levers for all masticatory muscles, larger incisors, and an increase in the shearing portion of m1 relative to the crushing portion. Most of these shape changes are similar to those described for PC3, which suggests that the female interspecific allometry bridges the bite force gap caused by sexual dimorphism in size. The changes to the female dentition suggest a shift in diet from crushing tough food items (e.g. aquatic invertebrates) towards slicing meat, which makes sense since these changes occur simultaneously with the common allometric trend (related to improved capabilities for killing larger vertebrate prey). However, as noted earlier, the increased shearing component is also advantageous for a piscivorous diet. Shape changes in male mandibles not observed in females seem to emphasize the common allometric trend (i.e., stronger killing bite at larger gapes) (Fig. S5D): a wider coronoid process for more muscle attachment, a dorsally displaced angular process to allow wider gapes, and mediolateral expansion of the corpus to increase its strength. Regarding their dentition, the opposite trend to females was observed (i.e., slightly smaller anterior teeth and a longer crushing molar portion), suggesting a larger durophagous component in the diet of larger males.

As expected, variation in mandible shape could be linked to potential dietary differences between European and American mink, and also between sexes. In summary, the results of the present study show that:

- American mink are better equipped for preying on terrestrial vertebrates, as they can achieve relatively larger gapes and their mandibles are able to produce larger forces during the killing bite (i.e., at the anterior teeth and with an open mouth).
- European mink, on the other hand, can produce relatively stronger bites at the carnassials, suggesting that they rely more on tougher prey and/or fish.
- Regardless of species and sex, morphological features in larger mink demonstrate increased capabilities for feeding on larger terrestrial prey (stronger killing bites and more robust anterior teeth and corpora to resist the stresses caused by struggling prey).
- Due to their larger size, male mink of both species have stronger bites than females at both the anterior teeth and the carnassials. However, with increasing size, females bridge the gap by developing relatively stronger bites overall while shifting their diet from tougher or harder prey (probably aquatic invertebrates) towards less mechanically demanding food items (e.g. terrestrial vertebrates and/or fish). In contrast, increasing size in males leads to even more specialization towards feeding on larger terrestrial prey while tough items become more relevant in their diets (probably crushing bones of small prey).

These findings confirm our original predictions based on previous results on cranial shape differences, but do they agree with observed dietary preferences in minks? Diet studies in American mink are numerous, and provide a wide picture of seasonal and regional variation^8,11^ as well as intraspecific dietary competition^6,7,12^. However, studies on European mink diet are scarcer^9,14^, particularly those comparing the sexes^13^. Additionally, a few studies have compared diets of sympatric European and American mink^10,15^. All these studies can be summarized as: A, male American mink favor medium-sized mammals and birds usually heavier than themselves; B, female American mink favor aquatic prey, but are displaced towards small mammals and birds when seasonal changes in prey availability shift the males’ diet towards aquatic prey; C, European mink favor aquatic prey, particularly fish and crayfish; but D, they are displaced towards amphibians and small mammals when sympatric with American mink. From these, our results on mandible shape variation support A and somewhat B and C, but provide no information on the interspecific competition scenario or on potential seasonal or local dietary differences. Additionally, there is no information on size-related dietary changes in either species that could validate our findings on sexual allometry in mandible shape. Thus, while mandible shape is very useful for identifying broad dietary indicators even between highly similar species, its ability to provide accurate information on their potential prey is limited.

As a final note on mink diets, our previous study on cranial shape^38^, suggested a gradient in muscle force (and potential dietary range) from female European mink to male American mink. Based on those results and studies on social interactions between and within species^35,46^, we hypothesized that competition between both mink species could be displacing female European mink towards narrower and poorer diets, which could affect their survivability and ability to successfully reproduce. Fortunately, the results of the present study not only propose that there might be less overlap in diets between species and sexes than suggested by dietary studies^7,10,13,15^, but also indicate that dietary competition seems to be higher for small terrestrial vertebrates, not aquatic prey (on which female European mink are particularly well equipped to feed).

## Material and methods

### Sample

We sampled a total of 170 adult mink mandible specimens: 58 European mink (Mlu) and 112 American mink (Nvi) (Table 3). As was observed in the cranial sample, we tried to capture as much morphological variation for each species as possible by including both female and male individuals from the three extant populations of European mink (northern Spain and southern France, the Danube Delta, and in some areas from Ukraine to northwestern Russia^37^), and from wild and feral American mink populations. Wild American mink came from their native range in North America, while feral specimens were sampled from different European countries to account for potential morphological variation due to the founder effect^47,48^. Species and sex data for some specimens were complemented with the results of a canonical variates analysis in cranial shape, whose correct classification rates were 100% for species and 93.8% for sex ^38^, Appendix S1.

**Table 3:** Sampled specimens.

Species	Females	Males	Total
European mink (Mlu)	22	36	58
American mink (Nvi)	56	56	112

The studied specimens belong to the collections of the Naturhistorisches Museum Basel (Switzerland), the Museum für Naturkunde (Berlin, Germany), the Bristol City Museum and Art Gallery (Bristol, United Kingdom), the Hungarian Natural History Museum (Budapest, Hungary), the National Museum of Scotland (Edinburgh, United Kingdom), the Muséum d’Histoire Naturelle de Genève (Switzerland), the Natural History Museum at the University of Oslo (Norway), the Muséum National d’Histoire Naturelle (Paris, France), the Estonian Museum of Natural History (Tallinn, Estonia), the Department of Archaeology at the University of York (United Kingdom), and the private collection of Dr Santiago Palazón (Flora and Fauna Service, Generalitat de Catalunya, Barcelona, Spain). Table S3 lists catalog numbers and other information (sex, locality, etc.) for each specimen.

The mandibles were imaged with the crania using micro-computed tomography (microCT) at the following facilities: Museum für Naturkunde (Berlin, Germany), the Natural History Museum at the University of Oslo (Norway), the Biomaterials Science Center at the University of Basel (Switzerland), the X-ray tomography facilities at the University of Bristol (United Kingdom), and ScanoMed Debrecen (Hungary). The same scanning and processing protocol as in Gálvez-López et al.^38^ was used.

### Landmark configuration

**Figure 3:**
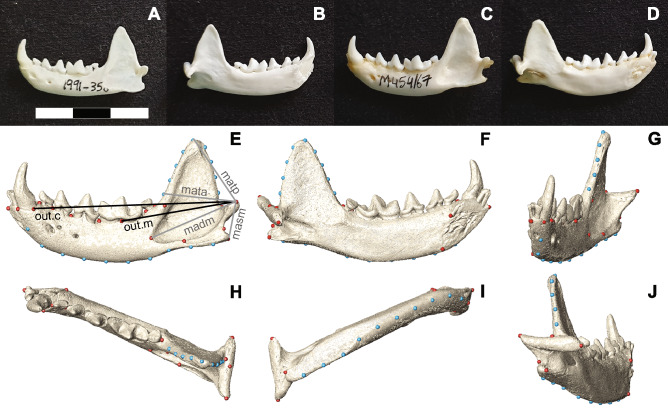
Mandible anatomy in minks (**A**–**D**) and landmark configuration used (**E**–**J**). European mink MNHN 1991–350 in lateral (**A**) and medial views (**B**). American mink NMS M454/67 in lateral (**C**) and medial views (**D**). Scale bar = 3 cm. See Table S3for additional information on both specimens. The landmark configuration is shown on the 3D mesh for the American mink above, in lateral (**E**), medial (**F**), cranial (**G**), dorsal (**H**), ventral (**I**), and caudal views (**J**). Red dots represent landmarks (as defined in Table 4), while blue dots represent semilandmarks along curves. Black lines in (**E**) represent the out-levers used in the biomechanical analyses, while grey lines represent the in-levers. Abbreviations: madm, lever arm of the deep masseter; masm, lever arm of the superficial masseter; mata, lever arm of the anterior temporalis; matp, lever arm of the posterior temporalis; out.c, out-lever at the canine; out.m, out-lever at the carnassial.

The 3D coordinates of 21 homologous landmarks and 19 semilandmarks were digitized on the left hemimandible of each specimen to quantify its morphological variation (Fig. 3). Landmark repeatability was assessed using the same protocol described in Gálvez-López et al.^38^, with the intraclass correlation coefficient^49,50^ amounting to 99.10% (i.e., landmark digitizing errors accounted for 0.9% of shape variation). The homologous landmarks (Table 4) were digitized in Avizo (version 7.1.0 for Windows, Visualization Sciences Group, Burlington, USA) together with two surface paths along curves. Using these surface paths, equidistant semilandmarks were placed along each curve: 9 on the coronoid process and 10 along the ventral margin of the hemimandible (Fig. 3).

**Table 4:** Landmark definitions.

Landmark	Definition
1	Left i1–right i1 cranial contact point
2	Cranialmost point of c alveolus
3	Lateralmost point of c alveolus
4	c––p2 lateral contact point
5	p2–p3 lateral contact point
6	p3–p4 lateral contact point
7	p4–m1 lateral contact point
8	Projection of the protocone cusp on the m1 alveolus
9	m1–m2 lateral contact point
10	Caudalmost point of m2
11	Most concave point between coronoid and condylar processes
12	Lateralmost point of condylar process
13	Medialmost point of condylar process
14	Dorsalmost point of mandibular condyle
15	Most concave point between condylar and angular processes
16	Most mediocaudal point of angular process
17	Most ventrocaudal point of symphyseal region
18	Most dorsocaudal point of symphyseal region
19	Projection of landmark 10 onto the border of the masseteric fossa
20	Cranialmost point of the masseteric fossa
21	Cranialmost point of the angular fossa on the border of the masseteric fossa

### Shape variation

All analyses were carried out in R (Version 4.0.3 for Windows^51^) within the RStudio environment (Version 1.3.1093 for Windows^52^), using the following packages: Arothron^53^, geomorph^54^, ggplot2^55^, Morpho^56^, magick^57^, rgl^58^, RRPP^59,60^, and stringr^61^. The code for all the analyses and computations can be found at https://git.io/JMEsw.

Landmark configurations of the whole sample were aligned using Generalized Procrustes Analysis (GPA)^62^, which standardizes the specimens in size, position and rotation. The centroid size (i.e., the square root of the sum of the squared distances of all points of the configuration to their centroid) of each configuration was calculated to use as size variable in subsequent analyses. Mandible shape variation was summarized in a 3D morphospace by performing a Principal Component Analysis (PCA) on the standardized configurations.

Potential interspecific differences in mandible shape between species and between sexes, as well as the relationship between size and shape, were assessed using Procrustes ANOVA^63^. Pairwise differences in mean Procrustes distances between groups (species, sexes) were also calculated and, where necessary, *p* values were adjusted using the Holm-Bonferroni method to account for multiple simultaneous comparisons^64^. Additionally, since factor interactions such as species with sex (interspecific differences in sexual dimorphism) and species with size (interspecific differences in shape allometry) were significant for cranial shape^38^, Procrustes ANOVA was also used to assess their effect on mandible shape. If an interaction was significant, the effect was further explored using phenotypic trajectory analysis (PTA), which identifies group differences in patterns of shape change in multivariate data^65^. Phenotypic trajectories along two-level variables, such as species and sex in this study, are defined by three parameters: location, magnitude and orientation. Testing for differences in location is equivalent to using pairwise comparisons between groups means (i.e., as described above), while differences in magnitude can be assessed comparing distances between group means. Finally, differences in orientation (i.e., whether both patterns of shape change follow a similar direction) can be tested by comparing the angle between both trajectories. The significance of all these tests was determined through randomization of residuals using permutation procedures (RRPP)^66,67^, performing 10,000 permutations for each analysis. Significance threshold was set at *p* < 0.05.

A similar protocol was used to analyze the relationship between species, sex and size and the main axes of shape variations (i.e., principal components, PCs) individually, but in this case using non-parametric ANOVA. This was preferred over traditional ANOVA because it is more robust in unbalanced, heteroscedastic designs such as the present study^68,69^. As above, significance (*p* < 0.05) was tested using RRPP with 10,000 permutations, adjusting *p* values where needed with the Holm-Bonferroni method.

### Muscle biomechanics

To provide a functional interpretation of shape variation in mink mandibles, the mechanical advantages (MA) of the main masticatory muscles (masseter, temporalis) at different bites were estimated from interlandmark distances in each specimen. MA represents the efficiency with which muscle force is translated into output force at the teeth, and can be estimated by dividing the lever arm of the muscle force (in-lever) by the lever arm of the resulting force at any particular tooth (out-lever)^70,71^. Note, however, that actual MA values are calculated using moment arms (not lever arms, as in this study), that is the perpendicular distance between the mandibular condyle and the vector running from the muscle origin to its insertion. Since lever arms have been used extensively in the literature to provide reasonable MA estimates^72^, and can be calculated independently of the cranium, they were preferred in this study.

Due to their complex anatomy and interspecific variability in attachment sites, there is no consensus on a common nomenclature for the masticatory muscles^25,73,74^. Thus, in this study a simplified functional approach was used to refer to masticatory muscles (Table 5). Note, however, that since the zygomaticomandibularis and masseter cannot be completely separated in mustelids^73,74^, we use masseter to refer to the resulting muscle complex.

**Table 5:** Muscle definitions.

Name	Origin	Insertion
**Temporalis**		
Anterior temporalis	Temporal fascia, cranial region of sagittal crest to postorbital process	Ventral half of cranial border of coronoid process
Posterior temporalis	Temporal fossa, nuchal crests and caudal region of the sagittal crest	Caudal border and medial surface of coronoid process
**Masseter**		
Superficial masseter	Ventral border of zygomatic arch, including cranial root and ventrolateral surface of caudal root	Ventral border of mandible, including angular fossa and lateral aspect of the angular process
Deep masseter	Medial surface of zygomatic arch, extending onto caudal root and preglenoid process	Masseteric fossa

In-levers were measured from the mandibular condyle (landmark L14) to the extreme points of their muscular insertion, as follows (Fig. 3E): anterior temporalis, base of the coronoid process (semilandmark SL22); posterior temporalis, tip of the coronoid process (SL26); superficial masseter, tip of the angular process (L16); deep masseter, cranialmost point of masseteric fossa (L20). Similarly, out-levers were measured from the mandibular condyle to the canine alveolus (L3) and to the m1 protocone (L8) (Fig. 3E). The former was used to represent the killing bite at the anterior teeth, and the latter to represent shearing and crushing bites at the carnassials. Since in carnivorans the anterior temporalis and deep masseter produce the main force at maximum gape (i.e., during the killing bite) and the posterior temporalis and superficial masseter are most effective with nearly closed jaws (i.e., at carnassial bites)^22^, only those two respective MAs were calculated for each bite.

Each set of lever arms and MAs was computed in R from the landmark configurations of the original specimens, and from the configurations associated with each of the first three PCs. As with PC scores, the effects of species, sex, size, and their interactions, were analyzed using non-parametric ANOVA tests (*p* < 0.05 significance threshold, 10,000 permutations, Holm-Bonferroni-adjusted *p* values).

## Supplementary Information

Supplementary Information 1.

Supplementary Information 2.

Supplementary Information 3.

Supplementary Information 4.

Supplementary Information 5.

Supplementary Information 6.

Supplementary Information 7.

Supplementary Information 8.

Supplementary Information 9.

## Data Availability

The data that support the findings of this study are available from the corresponding author upon reasonable request.
