# Supplementary material for: Mandible shape variation and feeding biomechanics in minks
Source: Sci Rep. 2022 Mar 23;12:4997. doi: 10.1038/s41598-022-08754-4 (PMC8943020; doi:10.1038/s41598-022-08754-4)
Supplement: Supplementary file 1 — Supplementary Information 1. [file 41598_2022_8754_MOESM1_ESM.pdf]

Supplementary Information

Table S1. Mechanical advantage values estimated for each specimen

Table S2. Results of the non-parametric ANOVAs on lever arms and mechanical advantages

Table S3. Measured specimens

Figure S1. Shape variation along PC1. The mesh corresponds to the specimen closest to the mean (*Neovison vison* NMS-M454/67), warped to the lowest PC1 score. The color pattern illustrates the regions of highest shape variation along PC1, with warm colors indicating a relative expansion with increasing PC scores, and cold colors representing a relative contraction.

Figure S2. Allometric trajectories. Interspecific and intraspecific regressions of PC scores on centroid size are presented for both PC1 (A, B) and PC2 (C, D), while the regressions shown for the MA of the anterior temporalis correspond to interspecific allometry (E) and interspecific sexual allometry (F). For interspecific regressions (A, C, F), dark grey denotes males and light grey females. For intraspecific allometry (B, D, E), blue circles represent European mink and pink squares American mink, while solid symbols are used for males and open symbols for females. Abbreviations as in Figure 1.

Figure S3. Shape variation along PC2. See Fig. S1 for details.

Figure S4. Shape variation along PC3. See Fig. S1 for details.

Figure S5. Trends in sexual dimorphism in mandible shape. Intraspecific sexual allometry (A), phenotypic trajectory analysis for species and sex (B), and allometric shape changes in mandible shape in females (C) and males (D). To visualize the trajectories in (B), multivariate data is summarized using a PCA on the fitted values, and the two first PCs are shown for each analysis (with percentage of total variance explained in parentheses). Symbols as in Figure 2, with mean scores for each group in black. In (C) and (D) the smallest specimen of each sex is presented in yellow, the largest in red.
