# Supplementary figures and images for: Mandible shape variation and feeding biomechanics in minks
Source: Sci Rep. 2022 Mar 23;12:4997. doi: 10.1038/s41598-022-08754-4 (PMC8943020; doi:10.1038/s41598-022-08754-4)

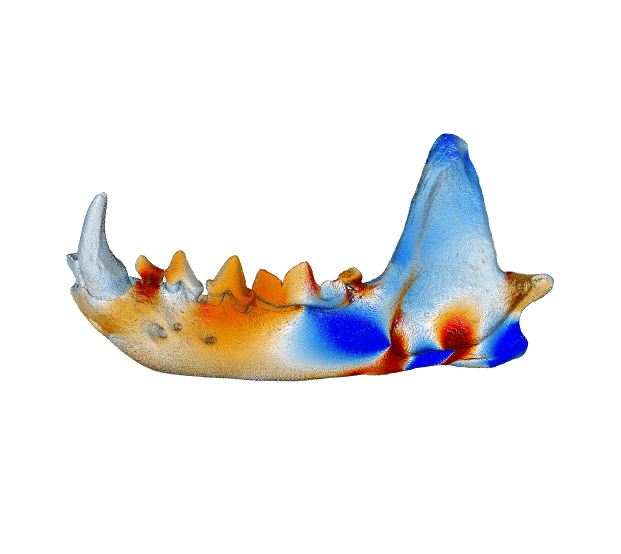

Supplement: Supplementary file 5 — Supplementary Information 5. [file 41598_2022_8754_MOESM5_ESM.gif]

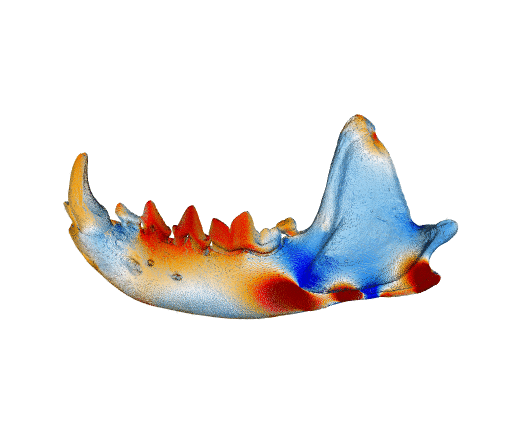

Supplement: Supplementary file 7 — Supplementary Information 7. [file 41598_2022_8754_MOESM7_ESM.gif]

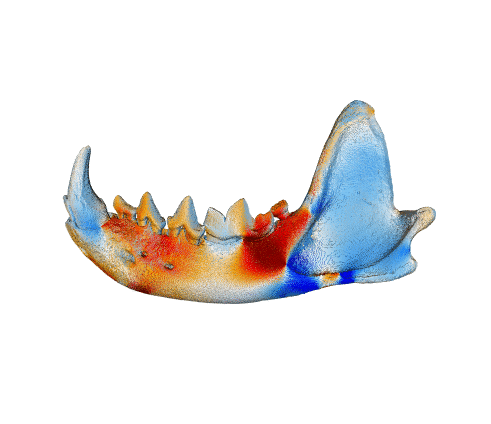

Supplement: Supplementary file 8 — Supplementary Information 8. [file 41598_2022_8754_MOESM8_ESM.gif]
